# Time from COVID-19 shutdown, gender-based violence exposure, and mental health outcomes among a state representative sample of California residents

**DOI:** 10.1016/j.eclinm.2020.100520

**Published:** 2020-08-24

**Authors:** Anita Raj, Nicole E. Johns, Kathryn M. Barker, Jay G. Silverman

**Affiliations:** a Center on Gender Equity and Health, Department of Medicine, University of California San Diego, San Diego, 9500 Gilman Drive #0507, La Jolla, CA 92093-0507, United States; b Education Studies, Division of Social Sciences, University of California San Diego, La Jolla, CA, United States

## Abstract

**Background:**

There is increasing evidence of the negative impact of the COVID-19 pandemic and resultant shutdowns on mental health. This issue may be of particular concern to those affected by intimate partner violence (IPV) and sexual violence.

**Methods:**

We conducted a cross-sectional analysis using data from a California state-representative online survey conducted in the two weeks following the state stay-at-home order, enacted March 19, 2020 (unweighted *N* = 2081). We conducted a series of multivariate multinomial logistic regressions to assess the associations between a) time since stay-at-home order and b) partner and sexual violence exposure ever with our outcomes of interest: depression and/or anxiety symptoms in the past two weeks. Covariates included demographics and social support.

**Findings:**

Nearly one in five (19•7%) respondents reported moderate or severe mental health symptoms in the past two weeks; 15•5% had a history of IPV and 10•1% had a history of sexual violence. In models adjusting for gender, partner and sexual violence history, and other demographics, time was significantly associated with greater mental health symptom severity, as were IPV and sexual violence. When we additionally adjusted for current social support, effects of time were lost and effects related to violence were slightly attenuated.

**Interpretation:**

Time under shutdown is associated with higher odds of depression and anxiety symptoms, and may be worse for those with a history of IPV. However, those with greater social support appear to have better capacity to withstand the mental health impacts of the pandemic. Social support programs, inclusive of those available virtually, may offer an important opportunity to help address increased mental health concerns we are seeing under the pandemic.

**Funding:**

Blue Shield Foundation of California Grant RP-1907–137. Bill and Melinda Gates Foundation OPP1179208.

**Research in context**

**Evidence before this study**

Evidence from China suggests increased mental health concerns as a consequence of the COVID-19 pandemic, but no research has examined whether time under the pandemic or a shutdown further elevate this risk. Additionally, there is indication of a rise in gender-based violence under the pandemic and resultant shutdowns, but this has not been analyzed in terms of how such violence or even histories of such violence may affect mental health.

**Added value of this study**

This study examines both time under shutdown/pandemic and exposure to partner and sexual violence ever as risk factors for severity in depression and/or anxiety symptoms in the past two weeks, with a state representative sample in California. It additionally examines whether current social support attenuates any observed effects of these exposure variables, to guide potential interventions at scale. Findings demonstrate higher than normal depression and/or anxiety symptoms in our sample under shutdown/pandemic conditions, and a significant association between time in days under shutdown/pandemic and severity of depression and/or anxiety symptoms. They also demonstrate elevated risk for poor mental health outcomes among those with a history of intimate partner violence (IPV), a concern disproportionately affecting women. However, findings also reveal that adjusting for social support eliminates findings related to time under shutdown and slightly attenuates findings related to IPV, suggesting the potential value of social support interventions implemented at scale to address mental health concerns under the pandemic and particularly for vulnerable populations.

**Implications of all the available evidence**

As COVID-19 impacts continue, it will be imperative for governments, health systems, and other support organizations to ensure mental health resource availability, both broadly and especially for these vulnerable populations. Social support systems and networks, including those delivered virtually, may be useful as we continue to contend with the pandemic and resultant social isolation.

Alt-text: Unlabelled box

## 1 Introduction

Global evidence documents the mental health impact of the COVID-19 pandemic and resultant government-enforced social distancing efforts, particularly the issues of depression and anxiety [1]. This issue may be of particular concern to those affected by intimate partner violence (IPV) and sexual violence, given the impact of these on mental health [2]. There are also indications of potential increase in such violence under COVID-19 related shutdowns [3] and as seen previously in other crisis contexts, such as earthquakes [4]. Isolation and feelings of helplessness may exacerbate these concerns in the face of the pandemic, as may financial stressors resulting from government shutdowns, particularly with ongoing time. Research has not examined associations of time under the COVID-19 pandemic and history of violence exposure with mental health outcomes. This study examines the association between time and self-reported depression and anxiety symptoms among a representative sample of California adults recruited over a two-week period in March 2020, at the start of the statewide government shutdown.

Our primary hypothesis of this study is that time – since shutdown and under the pandemic – will be associated with increased odds of reporting greater severity in depression and/or anxiety symptoms in the past two weeks, at a population level. Secondarily, we also consider whether history of IPV and sexual violence exposure, and financial status in the context of the shutdown, also independently affect severity of depression and/or anxiety symptoms, beyond that explained by time under the pandemic. Finally, we explore whether current social support attenuates or mitigates observed associations between time and depression and/or anxiety symptoms, as well as between our violence and income indicators and our outcome of interest.

## 2 Methods

### 2.1 Data source

We analyzed data from a representative sample of California residents aged 18 and older (*N* = 2081), surveyed on experiences of violence and mental health via an online survey implemented March 19th to March 27th, 2020 as part of the California Study on Violence Experiences Across the Lifespan 2020 (Cal-VEX 2020) [5]. The survey initiation date coincided with the first day of the governor-instituted statewide shutdown in response to the COVID-19 pandemic, and was around the time of statewide recognition of the presence of coronavirus in California and, in some places, identified community spread.

The Cal-VEX survey was developed by our team with inputs from an advisory board of experts on violence, and it was administered by NORC at the University of Chicago using their online probability panel (AmeriSpeak) and supplemented by additional nonprobability opt-in panels (Dynata and Lucid) to reach the desired sample size of 2000 individuals [5]. Statistical calibration was performed by NORC to combine these samples and create a survey-weighted final sample that is representative of the California adult population with regards to several key socio-demographics, including gender, age, race/ethnicity, income, education, employment status and region of the state (additional detail on data calibration and weighting methodology have been published elsewhere [6,7]). The recruitment rate for this study was 24%, and the response rate was 26%. These are standard for online panel surveys, which hover around 20–25% [8].

Our sample was generated from a general population sample of California adults age 18 and older selected from NORC's AmeriSpeak Panel. AmeriSpeak® is a probability-based panel designed to be representative of the US household population and is funded and operated by NORC at the University of Chicago. Randomly selected US households are sampled using area probability and address-based sampling, with a known, non-zero probability of selection from the NORC National Sample Frame. These sampled households are then contacted by US mail, telephone, and field interviewers (face to face). The panel provides sample coverage of approximately 97% of the U.S. household population. Those excluded from the sample include people with P.O. Box only addresses, some addresses not listed in the USPS Delivery Sequence File, and some newly constructed dwellings. While most AmeriSpeak households participate in surveys by web, non-internet households can participate in AmeriSpeak surveys by telephone. Households without conventional internet access but having web access via smartphones are allowed to participate in AmeriSpeak surveys by web. The AmeriSpeak panel sample was additionally supplemented with respondents from the Dynata and Lucid nonprobability online opt-in panels. Statistical calibration was conducted by NORC to combine these probability and non-probability samples to be representative of the state in terms of a pre-determined set of demographic and geographic characteristics.

To ensure the representativeness of the sample, our team compared the resultant sample to census and other government data. The study sample is representative of the adult California population with respect to gender, race/ethnicity, education level, employment status, income, age, and disability status. Our sample may slightly over-represent lesbian, gay, bisexual and other sexual minority individuals, and may slightly underrepresent foreign-born individuals and non-citizen residents. Findings should be considered in this light.

The NORC team contacted the participants to invite them into the online survey. The online survey took approximately 15 minutes to complete, and panelists were offered the cash equivalent of USD$2 for completing this survey. Survey participation was voluntary and allowed respondents to decline questions (outside of demographics) or stop the survey at any time. Participants in the survey panel agreed to privacy policies provided by NORC, and our research team only had access to completely anonymized data. Given the sensitivity of the survey items, a survey prompt was provided with the following text, “If you are experiencing distress or discomfort, see this website for services in the state https://victims.ca.gov/resources.aspx.” All research procedures were approved by both NORC/University of Chicago and the University of California San Diego Institutional Review Board (Project #191904XX).

### 2.2 Measures

Our primary independent variable of interest was time, measured by day of survey. By chance, our survey was initiated on March 19th, coinciding with the California state-wide stay at home order (in effect March 19, 2020), and ended on March 27th, allowing unique insight into potential effect of the pandemic on mental health. The resultant ‘time’ variable is a continuous variable of days.

Our dependent variable was severity of depression and/or anxiety, assessed via four items on the number of days the participant experienced depression and anxiety symptoms in the past two weeks, taken from the Patient Health Questionnaire-4 (PHQ-4) [9]. Anxiety was captured by two items, “Feeling nervous, anxious, or on edge” and “Not being able to stop or control worrying”. Depression was captured by two items, “Little interest or pleasure in doing things” and “Feeling down, depressed, or hopeless”. Response options were, “Not at all” = 0, “Several days” = 1, “More than half of the days” = 2, “Nearly every day” = 3. The severity of symptom score was created as stipulated by the PHQ-4 tool, by adding together the scores of each of the four items and categorizing scores as normal (0–2), mild (3–5), moderate (6–8), and severe (9–12). The Cronbach alpha for this measure was 0•81.

Covariates in our model included demographics (self-defined gender, monthly income categorized into wealth quintiles, employment, race/ethnicity, age, sexual orientation [gay, lesbian, bisexual, or straight], and disability), history of IPV and sexual violence, and social support. We assessed IPV via a series of items on whether the respondent had ever experienced physical violence (including being physically hurt or having a knife or gun pulled or used on them) or sexual abuse (including verbal sexual harassment, cyber sexual harassment, physically aggressive sexual harassment, quid pro quo sexual harassment, or forced sex) from a spouse or romantic partner. We created this measure for this survey based on prior research and expert input [5]. Cronbach alpha was 0•62. We assessed sexual violence by a single item on whether the respondent had every experienced “Forced sex - This can include someone forcing you to do a sexual act without your permission or one that you don't want to do (including while you are under the influence of alcohol or drugs).” This is a standard measure used in national surveys on sexual violence [10,11]. We assessed current social support via a single item from the Centers for Disease Control and Prevention's Behavioral Risk Surveillance System [12] which asked, “How often do you get the social and emotional support you need?” A four point response item was used: always, usually, rarely, or sometimes. Low reported social support from this item has previously been shown to be associated with poorer mental health outcomes [13].

### 2.3 Data analysis

We present frequency data on all key variables for the total sample and by gender, and we used chi-square analyses and t-tests to assess differences by gender on our variables of focus. We conducted a series of multinomial logistic regressions to assess associations between time and mental health symptom severity (normal, mild, moderate, severe): Model 1 adjusted only for time and gender. Model 2 additionally adjusted for IPV and sexual violence histories. Model 3 additionally adjusted for wealth quintile. Model 4 additionally adjusted for employment, race, age, sexual orientation, and disability. Model 5 additionally adjusted for social support, to see if this affected other observed associations. All analyses accounted for survey design and weighting to produce state-representative findings, and were conducted using STATA 15•1.

### 2.4 Role of funders

Funders had no role in the decision to develop these analyses or in the development of this manuscript for publication.

## 3 Results

The total sample of study participants was *N* = 2115, but our final analytic sample consisted of 2081 individuals, 1139 women and 942 men. Individuals missing information on the primary outcome (*n* = 22) were excluded, as were those identifying as transgender or other gender (*n* = 12) due to small cell sizes in our gender-stratified analyses.

Half of participants (53•5%) were in the normal range on our mental health outcome; 26•8% reported mild symptoms of depression and/or anxiety; 12•5% reported moderate symptoms; and 7•2% reported severe symptoms, in the past two weeks. (See Table 1.) We found that 15•5% and 10•1% of the sample reported a history of IPV and sexual violence ever, respectively. Women were significantly more likely than men to recent report depression and/or anxiety symptoms (51•6% vs 41•0%, *p* = 0•001), IPV (24•0% vs 6•4%, *p*<0•001) and sexual violence ever (16•1% vs 3•5%, *p*<0•001).

**Table 1:** Sample characteristics, total sample and by gender, of a state-representative sample of adults in California providing data from March 19 to 27, 2020 (*N* = 2081).

	Total N (%) or Mean (SD)*	Female N (%) or Mean (SD)*	Male N (%) or Mean (SD)*	Chi2 or *t*-test p-value, female vs male
*N*	*2081*	*1139*	*942*	
**Depression and/or Anxiety**				0.001
Normal	1095 (53•5%)	550 (48•4%)	545 (59•0%)	
Mild	545 (26•8%)	323 (29•5%)	222 (23•8%)	
Moderate	274 (12•5%)	153 (12•8%)	121 (12•2%)	
Severe	167 (7•2%)	113 (9•3%)	54 (4•9%)	
**Experiences of violence**				
IPV	347 (15•5%)	281 (24•0%)	66 (6•4%)	<0.001
Sexual violence	257 (10•1%)	216 (16•1%)	41 (3•5%)	<0.001
**Sociodemographics**				
*Income quintile*				<0.001
Quintile 1 [Less than $25,000]	405 (21•6%)	252 (24•6%)	153 (18•3%)	
Quintile 2 [$25,000-$49,999]	393 (18•6%)	241 (21•1%)	152 (16•0%)	
Quintile 3 [$50,000-$84,999]	483 (24•1%)	258 (21•8%)	225 (26•6%)	
Quintile 4 [$85,000-$124,999]	379 (17•6%)	206 (18•7%)	173 (16•4%)	
Quintile 5 [$125,000 or more]	421 (18•1%)	182 (13•7%)	239 (22•7%)	
*Employment*				0.02
Currently employed	1247 (56•6%)	646 (53•1%)	601 (60•5%)	
Not currently employed (includes retired)	834 (43•4%)	493 (46•9%)	341 (39•5%)	
*Race/ethnicity*				0.37
White	1176 (42•0%)	622 (42•4%)	554 (41•6%)	
Black	133 (5•6%)	70 (4•3%)	63 (7•0%)	
Asian	206 (12•9%)	108 (12•7%)	98 (13•1%)	
Hispanic	446 (32•9%)	267 (33•5%)	179 (32•2%)	
Other/multiple races	120 (6•6%)	72 (7•1%)	48 (6•1%)	
*Age*				0.24
18–29 years	431 (21•1%)	259 (23•5%)	172 (18•5%)	
30–44 years	606 (28•7%)	328 (28•2%)	278 (29•3%)	
45–59 years	453 (24•2%)	251 (23•7%)	202 (24•7%)	
v60+ years	591 (26•0%)	301 (24•6%)	290 (27•4%)	
*Sexual orientation*				0.001
Lesbian or Gay	86 (3•9%)	24 (1•9%)	62 (6•0%)	
Straight	1870 (90•5%)	1034 (91•5%)	836 (89•4%)	
Bisexual or Other Identity	124 (5•6%)	80 (6•6%)	44 (4•6%)	
*Disability Status*				0.80
Yes	535 (24•6%)	289 (24•3%)	246 (25•9%)	
No	1546 (75•4%)	850 (75•7%)	696 (75•0%)	
**Social support**				0.02
Rarely	292 (14•8%)	140 (11•8%)	152 (18•0%)	
Sometimes	459 (22•2%)	270 (23•4%)	189 (20•9%)	
Usually	802 (38•4%)	457 (40•8%)	345 (35•8%)	
Always	525 (24•6%)	271 (24•0%)	254 (25•3%)	
*Social support score*				0.83
Average score (range 1–4, higher = more support)	2•73 (0•99)	2•77 (0•97)	2•68 (1•01)	

⁎ Percentages, means, and standard deviations are survey weighted. Ns are unweighted.

Our initial models, those adjusting for gender (MODEL 1), gender and violence (MODEL 2), and gender, violence, and income (MODEL 3), all demonstrate significant positive association between time and depression and anxiety symptoms. (See Table 2.) For the initial models inclusive of our violence variables (MODEL 2 and MODEL 3), we also found IPV and sexual violence associated with mental health symptoms. Adjusted odds ratios [AORs] are presented. IPV ever was associated with greater odds of mild and moderate relative to normal mental health symptoms (e.g., MODEL 3 AORs: mild 1•78 [1•16–2•73]; moderate 2•86 [1•70–4•80]). Sexual violence ever was associated with increased odds of mild and severe relative to normal mental health symptoms (e.g., MODEL 3 AORs: mild 1•79 [1•11–2•88]; severe 2•88 [1•61–5•17]). All models (1–5) additionally found that male gender was negatively associated with having severe mental health symptoms.

**Table 2:** Logistic regression models assessing the association between time since government shutdown and depression and anxiety among a state-representative sample of adults in California providing data from March 19 to 27, 2020 (*N* = 2081).

	MODEL 1			MODEL 2			MODEL 3			MODEL 4			MODEL 5		
	Mild	Moderate	Severe	Mild	Moderate	Severe	Mild	Moderate	Severe	Mild	Moderate	Severe	Mild	Moderate	Severe
Date (continuous)	1•05* [1•00,1•10]	1•12⁎⁎⁎ [1•05,1•20]	1•09* [1•01,1•18]	1•05* [1•01,1•10]	1•13⁎⁎⁎ [1•05,1•21]	1•10* [1•01,1•20]	1•05* [1•01,1•10]	1•12⁎⁎⁎ [1•05,1•21]	1•09* [1•01,1•18]	1•02 [0•97,1•07]	1•07* [1•00,1•15]	1•02 [0•94,1•12]	1•01 [0•96,1•06]	1•06 [0•99,1•14]	0•99 [0•91,1•08]
Male (Ref Female)	0•66⁎⁎ [0•49,0•88]	0•77 [0•52,1•12]	0•43⁎⁎⁎ [0•26,0•70]	0•77 [0•57,1•04]	0•96 [0•64,1•45]	0•53* [0•32,0•89]	0•78 [0•57,1•05]	0•96 [0•64,1•45]	0•54* [0•32,0•91]	0•74 [0•54,1•02]	0•89 [0•59,1•34]	0•44⁎⁎ [0•26,0•75]	0•68* [0•50,0•93]	0•82 [0•55,1•24]	0•37⁎⁎⁎ [0•22,0•63]
IPV				1•76⁎⁎ [1•15,2•69]	2•82⁎⁎⁎ [1•69,4•73]	1•53 [0•88,2•68]	1•78⁎⁎ [1•16,2•73]	2•86⁎⁎⁎ [1•70,4•80]	1•43 [0•81,2•55]	1•64* [1•05,2•55]	2•60⁎⁎⁎ [1•53,4•41]	1•21 [0•66,2•24]	1•65* [1•06,2•57]	2•62⁎⁎⁎ [1•55,4•45]	1•27 [0•68,2•40]
Sexual Violence				1•78* [1•10,2•86]	1•50 [0•83,2•71]	2•86⁎⁎⁎ [1•62,5•06]	1•79* [1•11,2•88]	1•47 [0•81,2•68]	2•88⁎⁎⁎ [1•61,5•17]	1•60 [0•98,2•62]	1•35 [0•68,2•68]	1•90 [0•99,3•65]	1•51 [0•91,2•49]	1•27 [0•63,2•56]	1•59 [0•79,3•17]
Wealth Quintile (Ref: Lowest)															
2							1•11 [0•69,1•79]	0•78 [0•42,1•45]	1•35 [0•69,2•64]	1•41 [0•86,2•29]	1•41 [0•86,2•29]	1•93 [0•92,4•05]	1•4 [0•85,2•30]	1•06 [0•55,2•01]	1•99 [0•95,4•18]
3							0•81 [0•51,1•28]	0•78 [0•44,1•37]	1•29 [0•66,2•52]	1•00 [0•62,1•61]	1•00 [0•62,1•61]	1•91 [0•90,4•07]	1•07 [0•66,1•73]	1•17 [0•66,2•05]	2•21* [1•07,4•58]
4							0•9 [0•55,1•45]	0•73 [0•40,1•37]	0•45* [0•22,0•91]	1•21 [0•73,2•00]	1•21 [0•73,2•00]	0•75 [0•35,1•62]	1•35 [0•81,2•24]	1•29 [0•70,2•36]	1•00 [0•46,2•14]
5 (Highest Wealth Quintile)							1•06 [0•66,1•71]	0•98 [0•53,1•80]	0•46* [0•23,0•92]	1•33 [0•81,2•18]	1•33 [0•81,2•18]	0•71 [0•32,1•60]	1•44 [0•87,2•39]	1•57 [0•80,3•04]	0•92 [0•41,2•07]
Not Employed (Ref Employed)										1•02 [0•67,1•54]	1•02 [0•67,1•54]	0•91 [0•51,1•60]	1•1 [0•79,1•53]	0•99 [0•66,1•49]	0•87 [0•51,1•50]
Race/Ethnicity (Ref: White)															
Black										0•98 [0•53,1•81]	0•37* [0•15,0•90]	0•36* [0•14,0•91]	0•95 [0•49,1•85]	0•36* [0•15,0•88]	0•34* [0•13,0•90]
Asian										1•3 [0•86,1•99]	0•96 [0•53,1•73]	0•57 [0•18,1•81]	1•16 [0•76,1•78]	0•87 [0•47,1•60]	0•47 [0•16,1•36]
Hispanic										0•98 [0•67,1•44]	1•05 [0•63,1•75]	0•76 [0•43,1•36]	0•92 [0•63,1•34]	0•99 [0•59,1•65]	0•7 [0•38,1•28]
Other/multiple races										1•13 [0•65,1•98]	0•43* [0•19,0•98]	1•18 [0•56,2•46]	1•09 [0•62,1•89]	0•42* [0•19,0•94]	1•07 [0•51,2•26]
Age (Ref: 18–29 years)															
30–44 years										0•97 [0•64,1•48]	0•97 [0•64,1•48]	0•65 [0•35,1•22]	0•99 [0•65,1•51]	0•61 [0•36,1•04]	0•67 [0•35,1•27]
45–59 years										0•9 [0•56,1•45]	0•9 [0•56,1•45]	0•64 [0•33,1•23]	0•9 [0•56,1•45]	0•52* [0•30,0•92]	0•64 [0•33,1•25]
60+ years										0•46⁎⁎ [0•28,0•74]	0•46⁎⁎ [0•28,0•74]	0•11⁎⁎⁎ [0•05,0•23]	0•47⁎⁎ [0•29,0•77]	0•25⁎⁎⁎ [0•13,0•47]	0•12⁎⁎⁎ [0•05,0•25]
Sexual Orientation (Ref: Straight)															
Lesbian or Gay										1•57 [0•76,3•23]	1•22 [0•39,3•84]	4•23* [1•37,13•03]	1•63 [0•77,3•44]	1•3 [0•40,4•26]	4•84⁎⁎ [1•61,14•56]
Bisexual or Other										3•00⁎⁎⁎ [1•64,5•50]	1•75 [0•83,3•73]	2•84* [1•05,7•67]	2•79⁎⁎⁎ [1•53,5•06]	1•63 [0•77,3•46]	2•72* [1•08,6•82]
Disability										2•59⁎⁎⁎ [1•78,3•76]	5•09⁎⁎⁎ [3•30,7•85]	8•29⁎⁎⁎ [4•86,14•14]	2•65⁎⁎⁎ [1•82,3•86]	5•22⁎⁎⁎ [3•37,8•09]	8•85⁎⁎⁎ [5•15,15•21]
Social Support													0•68⁎⁎⁎ [0•58,0•79]	0•69⁎⁎⁎ [0•56,0•85]	0•44⁎⁎⁎ [0•34,0•58]

Referent group for all models is ‘normal’ mental health symptom severity.

All models account for survey weighting.

⁎ *p*<0.05.

⁎⁎ *p*<0.01.

⁎⁎⁎ *p*<0.001.

For our model adjusting for violence and all demographics (MODEL 4), we continued to see an association between each subsequent day of shutdown and mild, moderate, and severe symptoms of depression and/or anxiety in the past two weeks (MODEL 4 AORs: mild 1•05 [1•01–1•10]; moderate 1•12 [1•05–1•21]; severe 1•09 [1•01–1•18]). However, in this model, only IPV history was associated with higher odds for recent mental health symptoms (MODEL 4 AORs: mild 1•64 [1•05–2•55]; moderate 2•60 [1•53–4•41]); sexual violence findings were no longer significant. In terms of covariates, Black race/ethnicity or Other race/ethnicity (not Black, White, Asian, or Hispanic), relative to White, and older age (60+ years), relative to age 18–29 years, were negatively associated with reports of mental health symptoms. Minority sexual orientation relative to straight and disability relative to no disability were positively associated with having mental health symptoms in the past two weeks.

In our final adjusted model, which included current social support as well as demographics as covariates (MODEL 5), the association between time since shutdown and recent mental health symptoms was lost. However, IPV ever remained associated with greater odds of recent mild and moderate relative to normal mental health symptoms in this model (MODEL 5 AORs: mild 1•65 [1•06–2•57]; moderate 2•62 [1•55–4•45]), though findings were slightly attenuated. In terms of covariates, findings from the prior model were retained. Black and Other (not Black, White, Asian, or Hispanic) relative to White individuals and older (60+ years) relative to young adult (18–29 years) individuals were less likely to report mental health symptoms. Sexual minorities relative to straight individuals and those living with a disability compared to those without a disability were more likely to report having mental health symptoms in the past two weeks.

Findings related to income in our analyses were more complex across models. In our model inclusive of time, gender, violence, and income (MODEL 3), those in the higher and highest wealth quintiles, relative to those in the lowest, had lower odds of recent mental health symptoms (MODEL 5 higher wealth quintile AOR: severe 0•45 [0•22–0•91]; highest wealth quintile AOR: severe 0•46 [0•23–0•92]). In the model adjusting for all demographics (MODEL 4), significant findings were lost. However, in the model adjusting for all demographics and current social support (MODEL 5), findings as compared with MODEL 3 were altered. More specifically, higher and highest quintile findings were no longer significant, and elevated risk was seen for middle income individuals in terms of severe symptoms (MODEL 5 middle wealth quintile AOR: severe 2•21 [1•07–4•58]).

## 4 Discussion

Findings from this study demonstrate that approximately one in five people in this representative sample of California adults recruited during the first two weeks of pandemic shutdown report moderate to severe symptoms of depression and/or anxiety in the past two weeks, a higher prevalence than that seen in prior research with both general and patient populations under non-COVID-19 conditions [9,14,15]. These findings correspond with prior research suggesting increased odds of depression and anxiety due to COVID-19 and the resultant shutdown [1], and extend this work by highlighting that the odds of poor mental health, as indicated by symptoms, increase daily under shutdown conditions. They further indicate that those with a history of IPV or sexual violence, a concern disproportionately affecting women, are particularly vulnerable, findings seen in prior research as well [4]. Those with a history of IPV in particular appear to be at greater risk. Our IPV assessment does not allow for indication of recency, only history, and further research is needed to determine the relative roles of current versus history of IPV risk in contributing to poorer mental health outcomes. Indications that IPV may be increasing under shutdown conditions may be at play [3], but fears of isolation-related vulnerabilities for those with a history of such violence may also be a concern.

Importantly, however, findings from this study also show the potential value of active social support in mitigating risk for these symptoms, given loss of findings for time and somewhat attenuated findings in terms of IPV exposure after accounting for current social support. Socially marginalized groups such as sexual minorities and those living with a disability also reported greater odds of severity of mental health symptoms in the past two weeks, which again has been seen in prior research [16,17]. Disability in particular demonstrated a very strong association with severe depression and/or anxiety symptoms, even after adjusting for current social support. The pandemic and shutdown may be taking a greater toll on this population, due to their potential greater vulnerability to complications if coronavirus infection occurs and/or due to greater social and health vulnerabilities they may face generally. Overall, these findings suggest that already vulnerable populations may be greater risk for pandemic-related mental health concerns, but that social support may useful for management of these issues.

An additional finding from this study is the lower odds in recent severity of depression and/or anxiety symptoms for higher and highest income quintile groups related to the lowest income group, in models with time, violence exposure, and income. Such findings would suggest greater protection for higher income groups, possibly because of greater buffer against both viral exposure and financial stressors related to the pandemic and resultant shutdown. However, upon adjusting for demographics and social support, we see a notable change in the association between income and mental health outcomes, such that lower to middle-income groups have higher odds of poor mental health than the lowest income group. This unexpected finding may be a consequence of perceptions of greater vulnerability to the economic ramifications of the shutdown without safety net access (i.e., income-related welfare programs) or the greater representation of this population among essential workers, who may face greater risk for coronavirus exposure at work. Such findings highlight the importance of supports against financial stressors at this time, and for working populations.

While findings offer important insights, they should be considered in the light of certain study limitations. The effects of the pandemic are continuing, and we cannot know that our findings remain an accurate reflection of current circumstances; however, given the robust research evidence regarding mental health effects of the pandemic as described earlier, we believe findings likely remain relevant. Data are self-report and therefore subject to recall and social desirability biases. Findings are based on cross-sectional analyses and causality cannot be presumed from these findings. Longitudinal data, and data collected more regularly to account for seasonal and other variations that affect reports of depression and anxiety symptoms, would be important for future research to examine these issues. This would also allow for greater understanding of longer term effects of the pandemic and shutdown on these symptoms. Additionally, the survey was not designed to examine effects of the pandemic or time under shutdown, so we are limited in our assessments of both of these, the latter only allowing for a two week timeframe. We also cannot disaggregate time from shutdown and time under pandemic from these data. Our outcome covers a time preceding the stay at home order, but social distancing, and consequently social isolation, preceded the statewide shutdown order for many participants, as some counties had already received local shutdown orders [18] and some may have been reducing social contact prior to any order [19]. To consider this point, we analyzed severity of mental health symptoms by day of survey data collection, and compared this with state level data using similar indicators; findings confirm that, even by day one of data collection, we see higher than expected depression and/or anxiety symptoms. Prior statewide data from California indicate that approximately 5% reported moderate to severe anxiety symptoms and 2–3% reported moderate to severe depressive symptoms over a longer time period, past 30 days [15], whereas we found that 14% of our participants on day one of data collection reported moderate to severe depression and/or anxiety symptoms. Importantly, findings can only be understood in terms of symptoms and not mental health diagnosis.

We are also limited in our reliance on an online probability panel that facilitates engagement of a nationally representative sample, but participation rates were low. However, as noted above, these rates are typical of online studies [8,20]. Nonetheless, random sampling is the recommended approach to reduce potential biases inevitable in on-line rapid surveys that may reduce representative inclusion of those affected by mental health issues [21]. Hence, the current findings are likely not fully representative of those affected by depressive and anxiety-related symptoms in the current context of the pandemic, but this is likely yielding underrepresentation of the scope and scale of this concern. While this is a representative sample, via use of weighting procedures, it is also a convenient sample in the sense that it is an online survey, leaving it vulnerable to biases. NORC has taken steps to reduce some of the biases from typical online surveys as much as possible, including area probability and address-based recruitment and inclusion of non-internet and non-cell phone households in the survey panel sample. They also use a non-response follow-up campaign via the diverse contact information provided by respondents.

Ultimately, these findings add to the developing literature examining the health impacts of COVID-19, and suggest that COVID-19 and resultant shutdowns may be contributing to worsening mental health at a population level, with increasing risk over time. Moreover, individuals with a history of intimate partner or sexual violence, women, and other socially marginalized populations are disproportionately experiencing these mental health burdens, possibly because isolation and negative effects of isolation are worse for these groups. At the same time, the findings indicating the attenuation of time effects on mental health after accounting for current social support suggest the value of social support interventions to ameliorate mental health impacts. As COVID-19 impacts continue, it will be imperative for governments, health systems, and other support organizations to ensure mental health resource availability. This will be especially important for socially vulnerable populations. Social support systems and networks, including those delivered virtually, may be particularly useful as we continue to contend with the pandemic and resultant social isolation.

## Data sharing

Data from this study is freely available minus potentially identifiable demographic variables. If an individual is interested in receiving a copy of the dataset, they can send a request via email to geh@ucsd.edu. Please include in the subject line “request for Cal-VEX 2020 survey data.” Please include in the text of the email the purpose of the request and planned use of the data, including proposed research questions of interest.

## Funders

This project was funded through grants from the Blue Shield Foundation of California (Grant # RP-1907–137) and the Bill and Melinda Gates Foundation OPP1179208 This paper was developed independent of the study sponsors. They had no input into study design, data collection/analysis/interpretation, write-up of findings, or the decision to submit the paper for publication.

## Declaration of Competing Interest

The authors have no conflicts of interest to declare.

## Appendix Supplementary materials

Image, application 1
